# A Review of Marital Intimacy-Enhancing Interventions among Married Individuals

**DOI:** 10.5539/gjhs.v8n8p74

**Published:** 2015-12-17

**Authors:** Maryam Kardan-Souraki, Zeinab Hamzehgardeshi, Ismail Asadpour, Reza Ali Mohammadpour, Soghra Khani

**Affiliations:** 1 Faculty of Nasibeh Nursing and Midwifery, Mazandaran University of Medical Sciences, Sari, Iran; 2 Student Research Committee, Mazandaran University of Medical Sciences, Sari, Iran; 3 Department of Midwifery and Reproductive Health, Faculty of Nasibeh Nursing and Midwifery, Mazandaran University of Medical Sciences, Sari, Iran; 4 Research Center of Traditional Medicine, Mazandaran University of Medical Sciences, Sari, Iran; 5 Department of Counseling, Faculty of Psychology and Educational Sciences, Kharazmi University, Karaj, Iran; 6 Department of Biostatistics, Faculty of Health, Mazandaran University of Medical Sciences, Sari, Iran; 7 Research Center of Diabetes, Mazandaran University of Medical Sciences, Sari, Iran

**Keywords:** intervention, intimacy, marital

## Abstract

**Background:**

Lack of intimacy is currently the main concern rather than main concern of the experts in psychology and counseling. It is considered as one of the most important causes for divorce and as such to improve marital intimacy a great number of interventions have been proposed in the literature. Intimacy training and counseling make the couples take effective and successful steps to increase marital intimacy. No study has reviewed the interventions promoting marital intimacy after marriage. Thus, this review study aimed to classify the articles investigating the impact of interventional programs on marital intimacy after marriage.

**Search Methods:**

In April 2015, we performed a general search in Google Scholar search engines, and then we did an advanced search the databases of Science Direct, ProQuest, SID, Magiran, Irandoc, Pubmed, Scopus, Cochrane Library, and Psych info; Cumulative Index to Nursing and Allied Health Literature (CINAHL). Also, lists of the references of the relevant articles were reviewed for additional citations. Using Medical Subject Headings (MESH) keywords: Intervention (Clinical Trials, Non-Randomized Controlled Trials, Randomized Controlled Trials, Education), intimacy, marital (Marriage) and selected related articles to the study objective were from 1995 to April 2015. Clinical trials that evaluated one or more behavioral interventions to improve marital intimacy were reviewed in the study.

**Main Results:**

39 trials met the inclusion criteria. Eleven interventions had follow-up, and 28 interventions lacked follow-up. The quality evidence for 22 interventions was low, for 15 interventions moderate, and for one intervention was considered high. Findings from studies were categorized in 11 categories as the intimacy promoting interventions in dimensions of emotional, psychological, physical, sexual, temporal, communicational, social and recreational, aesthetic, spiritual, intellectual intimacy, and total intimacy.

**Authors’ Conclusions:**

Improving and promoting communication, problem solving, self-disclosure and empathic response skills and sexual education and counseling in the form of cognitive-behavioral techniques and based on religious and cultural context of each society, an effective step can be taken to enhance marital intimacy and strengthen family bonds and stability. Health care providers should consider which interventions are appropriate to the couple characteristics and their relationships.

## 1. Introduction

Marriage is a transient phase in one’s life and has always been emphasized as the paramount social ritual in order to meet the emotional needs of people (Dildar, Sitwat, & Yasin, 2013; Nayeri, Lotfi, & Noorani, 2014). In contemporary society, the incentives to marriage include the need to love and have intimate relationship with a partner, to have a companion in life, to satisfy psychological needs, and to increase joy (Soltani, Molazadeh, Mahmoodi, & Hosseini, 2013; Tavakol, Zarei, & Zeinali Pour, 2014).

Intimacy includes different meanings based on age, sex, education, and culture, and there is no consensus among researchers on the root concept of intimacy which makes its definition difficult (Martin & Tardif, 2014; Mitchell, 2007). Bagarozzi (2001) defines intimacy as proximity, similarity and a personal romantic or emotional communication that requires knowledge and understanding of another person to express thoughts and feelings (Bagarozzi, 2001).

Intimacy is strongly associated with the quality of couples’ life and is often referred to as a basic psychological need and one of the key characteristics of marital communication which impacts on marital adjustment and mental health, such as reducing the risk of depression, increasing happiness and well-being, and providing a useful satisfactory life of a person. Besides, it is a strong predictor of physical health, such as low level of diseases and impoverishment of diseases (Boden, Fischer, & Niehuis, 2010; Dandurand & Lafontaine, 2013; Moreira, Crespo, Pereira, & Canavarro, 2010; Nainian & Nik-Azin, 2013). In a study was shown that marital intimacy is effective on marital satisfaction (Greeff, Hildegarde, & Malherbe, 2001; Kim, 2013). Intimacy acts as a mediator between the effects of daily stress in relations between spouses (Harper, Schaalje, & Sandberg, 2000). There is a significant positive correlation between sexual satisfaction and marriage commitment with intimacy (Taghiyar, Mohammadi, & Zarie, 2015). In contrast, lack of intimacy is one of the most common causes of distress and collapse among couples, negatively impacting on relations between the couples and, thereby, leading to incompatibility and causes stress, and brings about psychological maladaptation, depression, and emotional disorders mental disorders (Duffey, Wooten, Lumadue, & Comstock, 2004; Kim, 2013; Yoo, Bartle-Haring, Day, & Gangamma, 2014). Dearth of intimacy is one of the most devastating problems that is difficult to be treated in the relationships (Whisman, Dixon, & Johnson, 1997). Weinberger et al. (2008) also showed that lack of intimacy in couples is the most important predictor of divorce in elderly (Weinberger, Hofstein, & Whitbourne, 2008). Thus, it can be stated that the consequences of failure in intimacy are manifold and physical divorce mainly arises from failure in intimacy (Duffey et al., 2004).

Therapists have described on various aspects that may negatively influence marital stability such as communication difficulty, unrealistic expectations from marriage and the spouse, lack of intimacy, and lack of expressing affection (Motavali, Ozgoli, Bakhtiari, & Alavimajd, 2010; Shahrestany, Doustkam, Rahbarda, & Mashhadi, 2013). Taking the fact for the granted that in many societies today family is the prominent source of comfort for people and taking the fact that in the modern society the family is faced with the challenges, the most important of which is the loss of marital intimacy, into account, an interventional program is helpful to prevent these problems and heighten intimacy (Farbod, Ghamari, & Majd, 2014). To enhance () intimacy in couples, educational approaches may support () (Oulia, Fatehizadeh, & Bahrami, 2006). It believed that Education and counseling per se may make the couples take effective and successful steps to increase marital intimacy (Hosseini Zand, SHafi Abadi, & Soudani, 2013). In Iran, some interventions are done to increase marital intimacy. For example, In a study was shown that training communication skills can enhance intimacy and quality in marital life (Farbod et al., 2014). Moreover, KhanjaniVeshki et al. (2012) concluded that sex education is effective in increasing sexual intimacy (Khanjani Veshki, Botlani, Shahsiah, & Sharifi, 2012). Duffey et al. (2004) also showed that sharing dreams and events between couples contributes to an increase in intimacy in couples (Duffey et al., 2004). According to the researcher’s search in the databases available, no study has reviewed the interventions promoting marital intimacy after marriage thus far. To address the latter the aim of this review is to classify the articles investigating the impact of interventional programs on marital intimacy after marriage.

## 2. Method

### 2.1 The Criteria Considered for This Review

#### 2.1.1 Type of Study

Clinical trials that evaluated one or more behavioral interventions to improve marital intimacy were reviewed in the study. Trials that focused on people with drug abuse and chronic health conditions, such as cancer, were excluded. The reason for their exclusion pertains to statistical population. This is because training and counseling to them were not proportionate to type of disorder in individuals and may not be applied for all spouses. Like educations and counselings that focused on people with breast cancer or prostate cancer. There was no other exclusion criterion.

#### 2.1.2 Type of Participants

Married men and women or couples.

#### 2.1.3 Type of Interventions

Interventions can have different formats such as verbal communication or written methods, individual or group counseling as well as using different types of technology, such as providing educational CDs. Intervention can be provided in a clinic or in the community and can target men, women, or couples. The comparison can be performed between the intervention under the study and another behavioral intervention, usual care or without any intervention.

#### 2.1.4 Type of the Measured Result

Our interest outcome was increasing intimacy. All trials that yielded this result were incorporated in the study.

#### 2.1.5 Type of Intimacy Assessment Tools

To evaluate interventions, different intimacy questionnaires can be applied such as:

Marital Intimacy Questionnaire Thompson and Walker Marital Intimacy Questionnaire (MIQ) (den Broucke & Vertommen (1995), Waring Intimacy Questionnaire (WIQ), Personal Assessment of Intimacy in Relationships inventory, Oulia’s Couples intimacy questionnaire, and Bagarozzi’s Marital Intimacy Needs Questionnaire.

### 2.2 Search Method

In April 2015, we performed a general search in Google Scholar search engines followed by an advanced search was done in the below databases:

ProQuest, Science Direct, SID, Irandoc, Magiran, Pubmed, Cochrane Library, Scopus, and Psych info; Cumulative Index to Nursing and Allied Health Literature (CINAHL).

Keywords were arranged based on Medical Subject Headings (MeSH) to search in Medline and based on non-mesh keywords in other databases including: Intervention (Clinical Trials, Non-Randomized Controlled Trials, Randomized Controlled Trials, and Education), intimacy and marital (Marriage).

Also, lists of the references of the relevant articles were reviewed for additional citations. Selected related articles to the study objective were from 1995 to April 2015.

### 2.3 Interventions Quality

The quality of evidence was evaluated. At first, the quality of the intervention design, implementation, and reports was evaluated. Quality of intervention downgraded for each of the following studies: 1) implementing intervention in less than two sessions, 2) the accuracy of reported interventional information for fewer than three items (Table 1), and 3) lack of follow-up (Lopez, Hiller, Grimes, & Chen, 2012; Lopez, Steiner, Grimes, & Schulz, 2013). The quality of the interventions evidence was recorded (Table 2) among the overall assessments of the quality of evidence (Table 3), the quality trials were considered high, then in the case of any of the following, one level of the quality of evidence was downgraded, A) lack of information on random sequence, allocation concealed, or lack of allocation concealed B) low quality interventions, and c) loss of more than 20% at follow-up. We considered a positive level for the studies that performed blinding procedures (Lopez et al., 2012).

**Table 1:** Intervention fidelity information

Study	Provider credentials	Provider education	Standardized delivery	Delivery adherence
Hosseinian (2012)	-----*	-----	1.5 hour session of communication skill based on Miller Theory	-----
Asadpour (2012)	Consultant	-----	12 sessions of emotionally focused couple therapy that each session lasted 2/5 hours	step by step and along with weekly assignments and regular exercises provided by consultants
Zarepour (2010)	-----	-----	The structure of sessions and trainings materials presented at each session were taken from Davison and Goldfried, Jacobson and Margolin, Miller et al., Bernstein and Bernstein, and was introduced during 6 weeks of one hour sessions	Training sessions were presented based on training curriculum
Salimi (2012)	-----	-----	sex education was presented in cognitive behavioral method during 6 sessions each lasted two hours	Training sessions were presented based on training curriculum
Nasr Isfahani (2013)	-----	-----	meaning - focused workshop in 10 sessions of 90 minutes	Once a week and based on the curriculum
Etemadi (2006)	consultant	-----	10 sessions of one-hour couple therapy based on cognitive behavioral techniques	Step by step and though weekly assignments
Ebrahimi (2011)	-----	-----	Communication enrichment program during 10 sessions of 1.5 hours	One session in a week
Rezaei (2013)	researcher	-----	7 sessions of Islamic lifestyle training with an emphasis on the family system	Twice a week sessions for 90 minutes each time
Shakarami (2014)	-----	-----	6 sessions of two hours sex education in the form of speech, asking questions, group discussion and presentation of assignments	Weekly program and based on the curriculum
Ghadam kheir (2013)	-----	-----	8 sessions of intervention based on intellectual-emotional behavior therapy	For eight weeks, every week for an hour and a half in groups
Mazlomi (2012)	-----	-----	Marriage enrichment preventive program designed by Mies and presented during 7 weeks	Every week one communication skill was taught to couples.
Etemadi (2014)	-----	-----	Eight sessions of an hour and a half of group training based on communication therapy approach	Weekly program and based on the curriculum
Hosseini Zand (2013)	researcher	-----	10 sessions of two hours of Islamic couple therapy training	Implemented once a week and in three stages
Shariatzadeh (2014)	researcher	-----	10 training sessions based on choice theory	-----
Oulia (2006)	-----	-----	6 sessions of 90-minute of marital life enrichment training	Sessions were hold step by step and weekly
BabaeiGarmkhani(2014)	-----	-----	8 sessions of 90-minute of cognitive behavioral group training	Twice a week and based on the curriculum
Botlani (2010)	-----	-----	8 attachment-based couple therapy sessions	Once a week and each session 90 minutes
Hosseini (2013)	-----	-----	8 sessions of 90-minute of solution-focused group counseling	Weekly and based on the curriculum
Nasr Isfahani (2010)	-----	-----	7 sessions of 90 minute of teaching concepts of choice theory	Once a week and based on the curriculum
Bahrami (2009)	offered under the counseling and leading of the supervising professor	-----	the 6 session 2-hr group enrichment program training	Weekly
Durana (1997)	therapist	Leaders are license mental health professional	The standard 4-month PAIRS format	Weekly or biweekly 3 hour sessions and 4 or 5 weekend workshop lasting about 21 hours.
Khanjani veshki (2012)	counselor	-----	6 sessions of sex education. Format and content identified for sessions	Sex training was presented step by step for men and women
Duffey (2004)	researcher	-----	offering of the intimacy-building, dream-sharing workshop and workbook used to the interventional group an intimacy-building and event-sharing workshop presented to the control treatment group	a four hour dream sharing workshop
Nayari (2014)	-----	-----	8 sessions of Transactional Analysis that each session lasted 1/5 hours	Training sessions were presented based on training curriculum
Hajian (2013)	-----	-----	An intensive course of solution-focused couples therapy was presented within six sessions that each session lasts 1.5 hours	Sessions for 2.6 months with giving assignments and feedbacks that presented based on Objectives listed for each visit.
Nasirnejhad karaj(2014)	-----	-----	Training positive thinking skills during 8 sessions that each session lasted 1/5 hours. Format and content identified for sessions	Weekly sessions
Mami (2015)	consultant	-----	ten sessions of 60 minutes couples therapy and cognitive-behavioral techniques	Weekly sessions
Farbod (2014)	-----	-----	12 sessions to enhance communication skills based marriage and family therapy	No information
Mohamadi (2013)	-----	-----	-----	-----
Coutta (2001)	A couple	A couple trained and husband had a Divinity degree with an emphasis in psychology and counseling.	A weekend marriage enrichment program With emphasis on Integrative Couple Therapy (ICT) developed by Neil Jacobson and Andrew Christensen.	Training session were presented based on training curriculum
Nasirnejhad [Tehran] (2014)	-----	-----	8 sessions of 1.5 hours training positive thinking skills. Format and content identified for sessions	twice a week
Sharifian (2011)	-----	-----	12 sessions of couple communication program (CCP) that each session lasted 2 hours	once a week
Hickmon (1997)	first author and a couple	The husband was in his final week of a masters’ degree program in Bible and Religion, training to be a family life minister.	In the Adventure group, Waring’s (1984) 8 components of marital intimacy in the design was used	two-day weekend
Soltani (2013)	-----	-----	8-10 sessions 120 minutes of emotionally focused couple therapy(EFCT)	EFCT has 3 stages and 9 steps.
Denton (2000)	therapist	Therapist was provided with 12 hr of training in emotion focused therapy that covered the theory and techniques of the approach.	8 sessions of emotion focused therapy (EFT) that each session lasted 50 minutes	Weekly
Karimi (2012)	-----	-----	-----	weekly
Yousefi (2014)	counselor	trained counselor in the Counseling Center	8 sessions of 1 hour based on Format and content identified for sessions	Two times a week.
Momeni Javid (2014)	-----	-----	9 sessions that each session lasted 1 hour. Format and content identified for sessions.	per week

* No information

**Table 2:** Quality of intervention* evidence

Study	Sessions <= 2	Having Follow-up	Intervention fidelity < 3 items	Quality1
Hosseinian (2012)	---	-1	-1	Low (-2)
Asadpour (2012)	---	-1	---	Moderate (-1)
Zarepour (2010)	---	-1	-1	Low (-2)
Salimi (2012)	----	-1	-1	Low (-2)
Nasr Isfahani (2013)	---	-1	-1	Low (-2)
Etemadi (2006)	---	-1	---	Moderate (-1)
Ebrahimi (2011)	---	---	-1	Moderate (-1)
Rezaei (2013)	---	-1	---	Moderate (-1)
Shakarami (2014)	---	---	-1	Moderate (-1)
Ghadam Kheir (2013)	---	-1	-1	Low (-2)
Mazlomi (2012)	---	-1	-1	Low (-2)
Etemadi (2014)	---	-1	-1	Low (-2)
Hosseini Zand (2013)	---	---	---	High (0)
Shariatzadeh (2014)	---	-1	-1	Low (-2)
Oulia (2006)	---	-1	-1	Low (-2)
Babaei Garmkhani (2014)	---	-1	-1	Low (-2)
Botlani (2010)	---	---	-1	Moderate (-1)
Hosseini (2013)	---	-1	-1	Low (-2)
Nasr Isfahani (2010)	---	-1	-1	Low (-2)
Bahrami (2009)	---	---	---	High (0)
Durana (1997)	---	---	---	High (0)
Khanjani Veshki (2012)	---	-1	----	Moderate (-1)
Duffey (2004)	-1	-1	----	Low (-2)
Nayari (2014)	---	---	-1	Moderate (-1)
Hajian (2013)	---	-1	-1	Low (-2)
Nasirnejhad Karaj (2014)	---	-1	-1	Low (-2)
Mami (2015)	---	-1	---	Moderate (-1)
Farbod (2014)	---	-1	-1	Low (-2)
Mohamadi (2013)	No information	-1	-1	Low (-2)
Coutta (2001)	No information	---	---	High (0)
Nasirnejhad Tehran (2014)	---	-1	-1	Low (-2)
Sharifian (2011)	---	---	-1	Moderate (-1)
Hickmon (1997)	---	-1	---	Moderate (-1)
Soltani (2013)	---	-1	-1	Low (-2)
Denton (2000)	---	-1	----	Moderate (-1)
Karimi (2012)	---	-1	-1	Low (-2)
Yousefi (2014)	---	---	---	High (0)
Momeni Javid (2014)	---	---	-1	Moderate (-1)

* Quality of the studies downgraded for each of the following studies: 1) implementing intervention in less than two sessions, 2) lack of follow-up, 3) the accuracy of reported interventional information for fewer than three items

**Table 3:** Quality of evidence*

Study	Randomization; allocation concealment	Losses > 20%	Blinding Quality of evidence1	Intervention quality	Quality of evidence1
Hosseinian (2012)	-1	No information	----	-1	Low(-2)
Asadpour (2012)	-1	No information	-----	-----	Moderate(-1)
Zarepour (2010)	-1	No information	---	-1	Low(-2)
Salimi (2012)	-1	No information	---	-1	Low(-2)
Nasr Isfahani (2013)	-1	No information	---	-1	Low(-2)
Etemadi (2006)	-1	No information	---	---	Moderate(-1)
Ebrahimi (2011)	-1	No information	---	---	Moderate(-1)
Rezaei (2013)	-1	No information	---	---	Moderate(-1)
Shakarami (2014)	-1	No information	---	---	Moderate(-1)
Ghadam Kheir (2013)	-1	No information	---	-1	Low(-2)
Mazlomi (2012)	-1	No information	---	-1	Low(-2)
Etemadi (2014)	-1	---	---	-1	Low(-2)
Hosseini Zand (2013)	-1	No information	---	---	Moderate(-1)
Shariatzadeh (2014)	-1	No information	---	-1	Low(-2)
Oulia (2006)	-1	---	---	-1	Low(-2)
Babaei Garmkhani (2014)	-1	No information	---	-1	Low(-2)
Botlani (2010)	-1	No information	---	---	Moderate(-1)
Hosseini (2013)	-1	No information	---	-1	Low(-2)
Nasr Isfahani (2010)	-1	No information	---	-1	Low(-2)
Bahrami (2009)	-1	No information	---	---	Moderate(-1)
Durana (1997)	-1	---	---	---	Moderate(-1)
Khanjani Veshki (2012)	-1	No information	---	---	Moderate(-1)
Duffey (2004)	-1	No information	---	-1	Low(-2)
Nayari (2014)	-1	No information	---	---	Moderate(-1)
Hajian (2013)	-1	No information	---	-1	Low(-2)
Nasirnejhad Karaj (2014)	-1	No information	---	-1	Low(-2)
Mami (2015)	-1	No information	---	---	Moderate(-1)
Farbod (2014)	-1	No information	---	-1	Low(-2)
Mohamadi (2013)	-1	No information	---	-1	Low(-2)
Coutta (2001)	-1	-1	---	---	Low(-2)
Nasirnejhad Tehran (2014)	-1	No information	---	-1	Low(-2)
Sharifian (2011)	-1	No information	---	---	Moderate(-1)
Hickmon (1997)	-1	No information	+1	---	High(0)
Soltani (2013)	-1	No information	---	-1	Low(-2)
Denton (2000)	-1	-1	---	---	Low(-2)
Karimi (2012)	-1	No information	---	-1	Low(-2)
Yousefi (2014)	-1	No information	---	---	Moderate(-1)
Momeni Javid (2014)	-1	No information	---	---	Moderate(-1)

* Quality could be high, moderate, low, or very low. We considered these RCTs to be high quality then downgraded a level for each of the following: A) lack of information on random sequence, allocation concealed, or lack of allocation concealed B) low quality interventions (Table 2), c) loss of more than 20% at follow-up. We upgraded one level for the studies that performed some blinding.

## 3. Search Results

**Figure 1:**
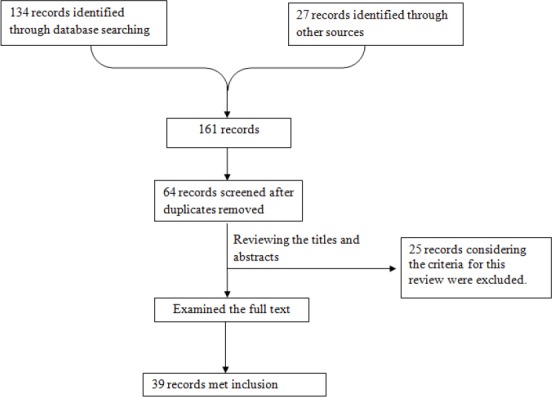
Result of the search

Sixty six sources were provided by the search from 1995 to April 2015. After reviewing the various titles and abstracts, 25 studies were excluded from review due to the lack of consideration of study criteria (the 25 studies that were excluded from review included 9 studies in the USA, 6 from Iran, 2 from Canada, 3 in each of Australia and Korea, whereas 1 was completed in each of England and the Netherlands, although they focusing on respondents with drug abuse and chronic health conditions) (Table 4). Finally, 39 trials met the inclusion criteria (Figure 1). The total number of participants was 1981 people, and the number of participants in each study was from 24 to 216 people. Average number of participants in each trial was 50.79 people. Thirty three studies were conducted in Iran, and the six others were conducted in America and Korea. Sixteen trials focused on women, 20 trials focused on couples, and 3 trials focused on men and women. Studies varied in provided educating content and format.

**Table 4:** Characteristics of excluded studies

Study	Reason for exclusion
Amber (2011)	Trial focused on couples with cancer
Leclerc	Trial focused on young adults with first psychological episode
Chambers (2014)	Trial focused on men with localised prostate cancer and their female partners
Zarei (2014)	Trial focused on spouses of war-disabled affiliated with markers and self sacrifices
Jun (2011)	Trial focused on Breast Cancer Survivors
Heather (2013)	Trial focused on men with localised prostate cancer
Robertson (2014)	Trial focused on Patients with prostate cancer and their partners
Reese (2012)	Trial focused on couple who had facing colorectal cancer.
Reese (2014)	Trial focused on couple who had facing colorectal cancer.
Julia (2009)	Trial focused on Patients with breast cancer
Jung (2005)	Trial focused on male patients with spinal cord injuries
Kerri (2012)	Trial focused on prostate cancer survivors (PCS) and their spouses
Manne (2004)	Trial focused on women with breast cancer and their partners
Nho (2013)	Trial focused on Women with Gynecologic Cancer and Their Husbands
Otto (2015)	Trial focused on women with breast cancer and their intimate partners
Gol (2013)	Trial focused on depressed patients
DeMarco (2009)	Trial focused on women living with or at risk for HIV.
Manne (2011)	Trial focused on Men Diagnosed with Prostate Cancer and Their Partners
Hummel (2015)	Trial focused on breast cancer survivors
Sidddons (2013)	Trial focused on men with localised prostate cancer.
Edward (1995)	Trial focused on depressed married women
Babapour Kheiroddin (2012)	Trial focused on chemical patient couples
Hamedi (2011)	Trial focused on addicted man and their Wives.
Sadrejahani (2009)	Trial focused on addicts and their wives
Kazemian (2013)	Trial focused on infertile Couples

Eleven interventions had follow-up (Bahrami, Oulia, & Isanezhad, 2009; Botlani, Ahmadi, Bahrami, Shahsiah, & Mohebbi, 2010; Coutta, 2001; Durana, 1997; Ebrahimi, Sanaei Zaker, & Nazari, 2011; Hosseini Zand et al., 2013; Momeni Javid, Soveyzi, & Mousavi, 2014; Nayeri et al., 2014; Shakarami, Davarniya, Zahrakar, & Gohari, 2014; Sharifian, Najafi, & Shaghaghi, 2011; Yousefi & Kiani, 2014) and 28 interventions lacked follow-up. Time of interventions was from one 4-hour workshop (Duffey) to 120-hour interventions for 4 to 5 months (Durana).

The quality of interventions was high in five studies, moderate in 13 studies was, and low in 20 studies. However, due to the limitations in the language in one study (the full text of the article was in Korean), it was not feasible to ensure the quality of the intervention (Table 2). The quality evidence was low for 22 interventions, moderate for 15 interventions, and high for one intervention (Table 3). Findings from studies were categorized in 11 categories as the intimacy promoting interventions in dimensions of emotional, psychological, physical, sexual, temporal, communicational, social and recreational, aesthetic, spiritual, intellectual intimacy, overall dimension, and total intimacy and are shown in Table 5.

**Table 5:** The intimacy-enhancing interventions in different dimensions

Dimension	Intervention	Authors
Emotional intimacy	Communication skill	Hosseinian (2012), Mazlomi (2012)
	Relationship Therapy	Etemadi (2014)
	Relationship enhancement program	Ebrahimi (2011)
	Marital enrichment	Oulia (2006), Bahrami (2009)
	Solution-focused couples therapy	Hajian (2013)
	Solution-Focused Group Counseling	Hosseini (2013)
	Cognitive-behaviour couple therapy	Etemadi (2006)
	Training of Islamic Lifestyle	Rezaei (2013)
	Emotional focused couple therapy	Soltani (2013), Asadpour (2012)
	(narrative therapy)	Mohamadi (2013)
Psychological intimacy	Communication skill	Hosseinian (2012), Mazlomi (2012)
	Relationship enhancement program	Ebrahimi (2011)
	Marital enrichment	Oulia (2006), Bahrami (2009)
	Solution-focused couples therapy	Hajian (2013)
	Solution-Focused Group Counseling	Hosseini (2013)
	Cognitive-behavior couple therapy	Etemadi (2006)
	Training of Islamic Lifestyle	Rezaei (2013)
	Emotional focused couple therapy	Soltani (2013), Asadpour (2012)
	Problem Solving Training	Zarepour (2010)
Physical intimacy	Communication skill	Hosseinian (2012), Mazlomi (2012)
	Relationship enhancement program	Ebrahimi (2011)
	Relationship Therapy	Etemadi (2014)
	Problem Solving Training	Zarepour (2010)
	Solution-focused couples therapy	Hajian (2013)
	Training of Islamic Lifestyle	Rezaei (2013)
	Emotional focused couple therapy	Soltani (2013), Asadpour (2012)
Sexual intimacy	Communication skill	Hosseinian (2012), Mazlomi (2012
	Relationship enhancement program	Ebrahimi (2011)
	Solution-focused couples therapy	Hajian (2013)
	Solution-Focused Group Counseling	Hosseini (2013)
	Cognitive-behavior couple therapy	Etemadi (2006)
	Training of Islamic Lifestyle	Rezaei (2013)
	Islamic couple therapy	Hosseini Zand (2013)
	Sex education	Shakarami (2014), Salimi (2012), Khanjani veshki (2012)
	Attachment-based couple therapy	Botlani (2010)
	Emotional focused couple therapy	Soltani (2013), Asadpour (2012)
	Training Positive Thinking	Nasiri Nejad (2014)
Temporal intimacy	Emotional focused couple therapy	Soltani (2013)
	Communication skill	Mazlomi (2012)
	Solution-Focused Group Counseling	Hosseini (2013)
Communicational intimacy	Relationship enhancement program	Ebrahimi (2011)
	Solution-focused couples therapy	Hajian (2013)
	Marital enrichment	Oulia (2006), Bahrami (2009)
	Training of Islamic Lifestyle	Rezaei (2013)
	Emotional focused couple therapy	Soltani (2013), Asadpour (2012)
	(narrative therapy)	Mohamadi (2013)
Social-Recreational intimacy	Communication skill	Hosseinian (2012), Mazlomi (2012)
	Relationship enhancement program	Ebrahimi (2011)
	Marital enrichment	Oulia (2006), Bahrami (2009)
	Emotionally focused couple therapy	Asadpour (2012)
	Training of Islamic Lifestyle	Rezaei (2013)
Aesthetic intimacy	Communication skill	Mazlomi (2012)
Spiritual intimacy	Communication skill	Hosseinian (2012), Mazlomi (2012)
	Relationship enhancement program	Ebrahimi (2011)
	Marital enrichment	Oulia (2006), Bahrami (2009)
	Emotionally focused couple therapy	Asadpour (2012)
	Training of Islamic Lifestyle	Rezaei (2013)
Intellectual intimacy	Communication skill	Hosseinian (2012), Mazlomi (2012)
	Relationship enhancement program	Ebrahimi (2011)
	Marital enrichment	Oulia (2006), Bahrami (2009)
	Emotional focused couple therapy	Soltani (2013), Asadpour (2012)
	Problem Solving Training	Zarepour (2010)
	Solution-focused couples therapy	Hajian (2013)
Total intimacy	Communication skill	Farbod (2014), Sharifian (2011), Karim I (2012)
	Problem Solving Training	Zarepour (2010)
	Training solution-focused couples therapy	Hosseini (2013)
	Dream sharing	Duffey (2004)
	Marital enrichment	Coutta(2001), Hickmon (1997)
	Training Positive Thinking	Nasirnejhad (2014)
	Cognitive- behavior couple therapy	Mami (2015), Etemadi (2006), BabaeiGarmkhani (2014)
	Meaning-centered training	Nasr Isfahani (2013)
	Choice theory training	Nasr Isfahani (2010)
	Rational - emotional behavioral therapy	Ghadam kheir (2013)
	Foot massage	Uhm (2010)
	Rogers Self Theory and Ellis Rational Theory	Yousefi (2014)
	Group training of transactional analysis	Nayeri (2014)
	Enhancing marital intimacy	Durana (1997)
	Emotion focused therapy	Denton (2000)

## 4. Discussion

### 4.1 Emotional Intimacy

emotional intimacy has been described as to share all the emotions, both positive and negative feelings with the spouse (Bagarozzi, 2001). Studies show that training and enriching the communication skills and communication therapy can contribute to the promotion of emotional intimacy (Ebrahimi et al., 2011; Etimadi, Jafari, & Seyah, 2014; Hosseinian, Yazdi, & Tabatabaei, 2012; Mazlomi, Dolatshahi, & Nazari, 2012). In these studies, participants were trained in some of the most important skills including conflict resolution by understanding the hidden needs and feelings of the spouse, understanding how to ask the needs and expectations, Identification of the impact of incorrect beliefs and expectations of spouses on the creation of conflicts and reduction of intimacy and active listening (Mazlomi et al., 2012), increasing self-awareness, knowing the spouse, getting familiar with each other’s needs and losses, renewing the memories of the past and improving the relations (Etimadi et al., 2014) and training communication skills based on Miller’s theory. So that in this plan, women were instructed the skills of speaking so as to convey information to the spouse, skills of listening, and skills of problem solving and planning in order to solve problems and identify effective communication styles (Hosseinian et al., 2012).

Solution-focused training plays an important role in increasing this dimension of intimacy. In this study, the couples were trained in six 90-minute training sessions to improve relationships and communication, evaluate the level of marital conflict and the nature of the problem, detect the chief complaint and define the problem, set a goal, examine solutions, formulate circles to find the solutions of the problems, and give the old and common solutions using intensive courses of couple therapy along with doing some homework in each session (Hajian & Mohammadi, 2013). The study showed that group counseling in the solution-focused method enhances the emotional intimacy (Hosseini, Majd, & GHamari, 2013). The other studies also stated that emotion-focused couple therapy can promote this dimension of intimacy (Asadpour, Nazari, Zaker, & Shaghaghi, 2012; Soltani et al., 2013). The 9-stage emotion-focused therapy of couples consists of description of the issues related to the conflict, identification of negative interaction circle that causes distress in couples, access unexplored emotions that are based on interactive conditions, formulation of the problem baced on emotion, like anger, disgust, fear, happiness, sadness and surprise. and attachment-focused needs, increase of the understanding of self emotions and personal needs that have been ignored, increase of accepting experiences of each spouse by the other party, creation of new ways of communicating, facilitation of expressing emotional needs and demands, facilitation of the development of new solutions for old problems and finally integration and reinforcement of new situations (Asadpour et al., 2012; Soltani et al., 2013).

Etemadi et al. (2006) showed in their study that the use of cognitive-behavioral techniques can promote emotional intimacy. In the cognitive behavioral techniques, participants were studied in terms of having unrealistic expectations and beliefs about intimacy and sexual relationships and the destructive effects of such behaviors on feelings, eliminating misunderstandings arising from misconceptions or different understanding, assessing the problems associated with the message sender and receiver and training communication skills, creating empathic understanding and active listening comprehension skills, training problem solving skills, and exploring the conflicts between spouses weekly and along with assignments (Etemadi, Navvabi Nezhad, Ahmadi, & Farzad, 2006). Other interventions to promote emotional intimacy can be pointed out as narrative therapy (Mohammadi, Sohrabi, & Aghdam, 2013), the Islamic lifestyle (Rezaei et al., 2013), and enriching the marital life (Bahrami et al., 2009; Oulia et al., 2006).

### 4.2 Psychological Intimacy

Psychological intimacy involves sharing personal issues, information, hopes, fears, desires, and feelings about the self with a spouse (Bagarozzi, 2001). The study of Ebrahimi et al. (2011) showed that enriching communication plays an important role in enhancing this dimension of intimacy. Here, in the relationship enrichment program, expressive skills, empathic listening, correct simultaneous way of speaking and listening and comparing it with the non-skilled dialogue, conflict resolution skill, self -change skill, and the skill of helping the spouse to change of the participants of the study were investigated (Ebrahimi et al., 2011). The studies of Hosseinian et al. (2012) and Mazlomi et al. (2012) came across results in line with the study of Ebrahimi (2011) and showed that enriching communication skills contributes to a rise in intimacy. Education of problem-solving skill among couples leads to increasing psychological intimacy (Zarepour, 2010), that is in line with the study of Hajian (2013) (Hajian & Mohammadi, 2013). Zarepour performed the education of problem-solving skill in order to take a positive and optimistic attitude towards the problem and the ability of the couples to deal with it, identify problems and obstacles to solve the problem, identify realistic objectives agreed by the couples, evaluate each solution and select the best solution, and implement the selected solution in the real life (Zarepour, 2010). Other interventions that can promote the psychological intimacy can be emotion focused couple therapy skill (Asadpour et al., 2012; Soltani et al., 2013) and the Islamic lifestyle approach. The Islamic life style approach is based on Islamic rules and principles more expounded on in the following sections. (Rezaei et al., 2013)

### 4.3 Physical Intimacy

Physical intimacy is the partner’s need to physical contact such as hugging, holding hands and non-sexual touch (Bagarozzi, 2001). Education of problem-solving skill is effective in increasing this dimension of intimacy (Zarepour, 2010). The studies of Hajian (2013) and Hosseini (2013) were consistent with the study of Zarepour (2010) and showed that solution-focused training leads to increasing physical intimacy (Hajian & Mohammadi, 2013; Hosseini et al., 2013). Emotion-focused couple therapy can promote the physical intimacy of the couples (Asadpour et al., 2012). The study of Soltani (2013) is also in line with the study of Asadpour (Soltani et al., 2013). Other interventions that can promote the physical intimacy can be communication enriching (Ebrahimi et al., 2011; Etimadi et al., 2014; Hosseinian et al., 2012; Mazlomi et al., 2012) and Islamic lifestyle training (Rezaei et al., 2013).

### 4.4 Sexual Intimacy

Sexual intimacy involves the expression of thoughts, feelings, and desires that have sexual nature and are planned to arouse sexual stimulation and sexual satisfaction (Bagarozzi, 2001). One of the ways to increase sexual intimacy is to present sex education to couples (Shakarami et al., 2014). Education and counseling in sexual dimension cause the couples to get enough awareness in this field and take effective steps to deal with their sexual problems and promote sexual intimacy (Zand et al., 2013). Sex education based on cognitive-behavioral techniques is effective to improve sexual intimacy (Veshki et al., 2012). Sex education increases sexual intimacy (Salimi & Fatehizadeh, 2012; Shakarami et al., 2014). In sex education, participants become familiar with physiology and sexual behavior and also receive education on topics such as modifying the myths about sexual matters, shaping the sexual intimacy and appropriate sexual techniques, and getting familiar with some of the most common sexual disorders (Shakarami et al., 2014). In addition, in the study of Salimi (2012), participants received trainings such as relaxation and fantasy skill, attention and awareness of the Sensory symptoms, expression of emotion and self -sexual expression, establishment of sexual intimacy, increase of positive self-talk, communication skill, increase of positive interactions, and problem solving (Salimi & Fatehizadeh, 2012).

Couples’ communication skill improvement increased sexual intimacy (Mazlomi et al., 2012). The study of Hosseinian (2012) and Ebrahimi (2011) are in line with the study of Mazlomi (2012) (Ebrahimi et al., 2011; Hosseinian et al., 2012). While Etemadi (2014) showed that communication therapy does not improve the sexual intimacy (Etimadi et al., 2014). Nasiri Nejad (2014) found that educating positive thinking is conducive to sexual function and sexual intimacy of the spouses. In this study, participants were first familiarized with the need to positive thinking, different coping styles, and the ways to forming thinking and attitudes. The participants were then familiarized with their negative thoughts and modification techniques and also with positive thinking and its effect on the life based on cognitive-behavioral technique. Applying the ABC theory of, (The ABC Model A major aid in cognitive therapy is what Albert Ellis (1957) called the ABC Technique of Irrational Beliefs. The first three steps analyze the process by which a person has developed irrational beliefs including: A - Activating Event or objective situation, B - Beliefs and C - Consequence. Ellis believes that it is not the activating event (A) that causes negative emotional and behavioral consequences (C). Rather, a person interprets these events unrealistically and, therefore, has an irrational belief system (B) that helps cause the consequences (C) (McLeod, 2008)). The participants were trained in forming positive thoughts for example: 1) Im responsible and in control of my life. 2) Circumstances are what they are, but I can choose my attitude towards them. And 3) Every challenge that comes along is an opportunity to learn and grow. 4) I am getting better every day.). Training techniques to stop negative thoughts, boosting self confidence, and adding laughter and sports to life were other positive thinking techniques which were relied upon (Nejad, Nazari, & Bahrainian, 2014). Other interventions to promote sexual intimacy can be solution-focused training (Hajian & Mohammadi, 2013; Hosseini et al., 2013), use of cognitive-behavioral techniques (Etemadi et al., 2006), the Islamic lifestyle (Rezaei et al., 2013), couple therapy (Zand et al., 2013), couple therapy based on attachment (Botlani et al., 2010) and emotion-focused couple therapy (Asadpour et al., 2012; Soltani et al., 2013).

### 4.5 Temporal Intimacy

Temporal intimacy indicates the extent to which couples tend to spend their daily time with their spouses on intimate activities (Bagarozzi, 2001). The study of Soltani et al. (2013) showed that emotion-focused couple therapy can promote this dimension of (Soltani et al., 2013). The first hypotheses on excitement-based treatments contend that the most effective factor in creating and maintaining marital intimacy is the type of the existing chain of excitement. Johnson (2004) predicts that excitement-based treatment (emphasizing sympathy, self-expression, deep understanding of one’s self needs and the partner’s needs, acceptance, expression of ideas and feelings and creation of an emotional environment, all of which are considered as essential elements in an intimate relationship) can play a powerful role in increasing intimacy in couples (Hamedi, Abadi, Navabinejad, & Delavar, 2013). Other interventions that can be pointed out to increase intimacy are communication skill training (Mazlomi et al., 2012) and solution-focused group counseling, in Solution-based treatment focuses on the activities both of the spouses enjoy and encourages them to do those activities again. Recommending the couple to walk and have recreation together without the presence children can be significant help to them in an optimal use of their time (Hosseini et al., 2013).

### 4.6 Communication Intimacy

Communication intimacy is defined as the creation of a relationship with respect, commitment, and positive emotions in such a way that the spouses feel valued and respected in this communication (Oulia et al., 2006). Bahrami (2009) carried out a study with the purpose of enriching marital life, and the intervention group was trained to have intimacy, improve sex issues, manage household, restructure cognition, and learn conflict resolution skill. The results showed that training marital life enrichment enhances communication intimacy (Bahrami et al., 2009). The results of the study of Oulia (2006) are consistent with the above study (Oulia et al., 2006). Hajian (2013) reported that group solution-focused training is related to promoting communication intimacy by promoting intimacy (Hajian & Mohammadi, 2013). Other study also showed that communication enrichment is associated with increased communication intimacy (Ebrahimi et al., 2011; Hosseinian et al., 2012).

Moreover, Soltani (2013) stated that the emotion-focused therapy increased communication intimacy (Soltani et al., 2013) that the study of Asadpour (2012) is consistent with the above study (Asadpour et al., 2012). Other interventions promoting communication intimacy include narrative therapy (Mohammadi et al., 2013) and Islamic lifestyle (Rezaei et al., 2013).

### 4.7 Social-Recreational Intimacy

Social recreational intimacy requires involving the spouse in responsibilities, passing holidays, enjoyable activities and leisure time, and expressing experiences and daily events (Bagarozzi, 2001). It was shown in the studies that communication skill training increases this dimension of intimacy (Ebrahimi et al., 2011; Hosseinian et al., 2012; Mazlomi et al., 2012); however, Etemadi (2014) showed that communication therapy has no positive effect in promoting social recreational intimacy (Etimadi et al., 2014). Asadpour (2012) demonstrated that emotion-focused couple therapy can promote social recreational intimacy while Soltani (2013) stated that emotion-focused couple therapy has no significant effect on increasing this type of intimacy (Asadpour et al., 2012; Soltani et al., 2013). In emotion-focused couple therapy, the first hypotheses on excitement-based treatments contend that the most effective factor in creating and maintaining marital intimacy is the type of the existing chain of excitement. Johnson (2004) predicts that excitement-based treatment (emphasizing sympathy, self-expression, deep understanding of one’s self needs and the partner’s needs, acceptance, expression of ideas and feelings and creation of an emotional environment, all of which are considered as essential elements in an intimate relationship) can play a powerful role in increasing intimacy in couples (Hamedi et al., 2013). Rezaei (2013) showed in his study that the Islamic lifestyle training increases recreational intimacy between spouses and that a summary of Islamic lifestyle training content includes the definition of marital intimacy, expression of couples’ expectations from their marital life, Islam’s idea about intimacy and the ways to increase it, verbal and nonverbal communications of the spouses, role of forgiveness in the conjugal life, guidance in order to enhance the relationships among couples, rights of spouses towards each other and respecting the boundaries in the families, sex customs in Islam and respect for privacy in sexual relationships, procedures of creating peace in the family, and methods of conflict resolution in the family (Rezaei et al., 2013).

### 4.8 Aesthetic Intimacy

Aesthetic intimacy needs sharing feelings, thoughts and beliefs that are Beautiful exciting in one’s opinion (Aesthetic intimacy needs sharing feelings, thoughts, and beliefs which are beautiful excitements in one’s opinion, such as wonders of nature and the cosmos, music, art, poetry, etc. (Bagarozzi, 2001). Mazlomi (2012) demonstrated in that communication skill training can promote the aesthetic intimacy (Mazlomi et al., 2012), while the study of Etemadi (2006), which was performed to evaluate the effect of cognitive behavioral techniques training on intimacy and the intervention group, was trained skills of communication, problem solving, and conflict. Besides, cognitive factors showed that the above skills do not have any effects on improving this dimension of intimacy (Etemadi et al., 2006).

### 4.9 Spiritual Intimacy

Religious intimacy is described as to express your thoughts, feelings, beliefs and experiences about religion, supernatural issues, moral values, life after death, and the relationship with God for your spouse (Bagarozzi, 2001). Mazlomi et al. (2012) showed that promoting communication skill of couples increases the intimacy in this dimension (Mazlomi et al., 2012). The other studies are in line with the study of Mazlomi (2012) (Ebrahimi et al., 2011; Hosseinian et al., 2012), while Etemadi et al. (2014) reported that communication therapy does not improve religious intimacy (Etimadi et al., 2014). Asadpour (2012) also showed that emotion-focused couple therapy can promote religious intimacy; on the other hand Soltani (2013) showed that emotion-focused couple therapy has no significant effect on increasing this dimension of intimacy (Asadpour et al., 2012; Soltani et al., 2013). Also Oulia (2006) reported that the marital life enrichment can promote religious intimacy that is in line with the study of Bahrami (2009) (Bahrami et al., 2009; Oulia et al., 2006). The results of the study of Rezaei et al. (2013) also showed that religious intimacy is effective in improving the Islamic lifestyle (Rezaei et al., 2013).

### 4.10 Intellectual Intimacy

Intellectual intimacy is the need to transfer and restate important thoughts and beliefs with the spouse (Bagarozzi, 2001). The study showed that enrichment of marital life can promote the intellectual intimacy (Oulia et al., 2006). The other study is in line with the above study (Bahrami et al., 2009). The results of the study of Mazlomi et al. (2012) stated that improving the communication skill of couples increases intellectual intimacy (Mazlomi et al., 2012). The other studies are in line with the study of Mazlomi (2012) (Ebrahimi et al., 2011; Hosseinian et al., 2012), while Etemadi et al. (2014) showed that the communication therapy has no effect on the promotion of intellectual intimacy (Etimadi et al., 2014). Zarepour (2010) showed that training problem-solving skill leads to the improvement of intellectual intimacy between the couples (Zarepour, 2010). Solution-focused training of the couples can promote this aspect of intimacy (Hajian & Mohammadi, 2013). Emotion focused therapy increases this dimension of intimacy (Soltani et al., 2013), which is in line with the study of Asadpour et al. (2012).

### 4.11 Total Intimacy

Momeni Javid et al. (2014) reported that training marital life promoting skills has an effective role to improve marital intimacy (Javid et al., 2014). The results of the other studies are in line with this study (Farbod et al., 2014; Karimi, Hasani, Soltani, Dalvand, & Zohdi, 2012; Sharifian et al., 2011). Moreover, Zarepour (2010) showed that promoting problem-solving skill in couples is associated with increasing the overall intimacy that the study of Hosseini (2013) is in line with it (Hosseini et al., 2013; Zarepour, 2010). Sharing dreams and events can promote total intimacy (Duffey et al., 2004). Marital life enrichment of couples increases the intimacy that is in line with the study of Hickmon (1997) (Coutta, 2001; Hickmon Jr, Protinsky, & Singh, 1997). Enrichment of intimacy promoting program increases marital intimacy (Durana, 1997). The study reported that the use of cognitive-behavioral techniques enhances the intimacy of the couples which is consistent with the other studies (Babaei Garmkhani, Madani, & Lavasani, 2014; Etemadi et al., 2006; Mami, Roohandeh, & Kahareh, 2015). Emotion focused therapy can promote intimacy (Denton, Burleson, Clark, Rodriguez, & Hobbs, 2000) and emotional intellectual behavioral therapy can promote intimacy (Ghadam kheir, Ghamari Givi, Niloofar, & Sepehri Shamlo, 2013). Education of choice theory concepts increases marital intimacy (Nasr Isfahani, 2010), while the results of the study of Shariatzadeh (2014) suggested that the effect of training choice theory in group method was not significant to increase marital intimacy (Shariatzadeh, Tabrizi, & Ahghar, 2014). Other intimacy promoting interventions include positive thinking (Nasiri Nejad, Tork, Zahedi Rad, Nazari, & Korivand, 2014), meaning focused training (N. Nasr Isfahani, Etemadi, & Shafie Abadi, 2013) foot massage (Uhm, 2010), Rogers and Ellis psychotherapy (Yousefi & Kiani, 2014) and group training of transactional analysis (Nayeri et al., 2014).

## 5. Conclusion

Overall, it can be stated from reviews that since intimacy involves the exchange of deep feelings and personal and private thoughts, promoting communication skill can play an important role in promoting intimacy in couples. In addition, according to the point that problem solving skill helps couples to evaluate the solutions to their problems and find more sense of cooperation and empathy, it could be accounted for as one of the most important factors for increasing the agreement and intimacy in couples. The depth of intimacy that people understand in their communications depends on their ability to handle correct, effective, and clear communications with the expression of feelings, needs, and desires.

Based on the results, it can be expressed that self-disclosure and empathic response can also increase intimacy because; when people trust each other and share their thoughts, feelings, and internal reality, it helps them strengthen the intimate communication in couples. Also, it can be concluded from studies that sex education and counseling helps the couples gain sufficient knowledge in this area and take effective steps to deal with sexual problems and enhance their intimacy. Generally, by promoting communication, problem solving, self-disclosure, empathic response skills, and sexual education and counseling in the form of cognitive-behavioral techniques, based on religious and cultural context of each society, an effective step can be taken to enhance marital intimacy and strengthen family bonds and stability. Therefore, it is recommended to provide and present counseling training packages to increase marital intimacy tailored to the cultural context of the society.

### 5.1 Implications for Practice

The majority of the interventions that promoted marital intimacy were quasi experimental. Interventions need to be adapted to other environments and tested again. Health care providers should consider which interventions are appropriate to the couple characteristics and their relationships and then use them.

### 5.2 Application in Research

The quality of many of the interventions was low and medium and did not have enough follow-up. The researchers need to design high-quality clinical trials with long-term follow-up period appropriate to the setting and resources. Also the processes of randomization and concealments are applied in designing interventions. It is also recommended that researchers measure effectiveness of interventions in raising marital intimacy.
